# Molecular characterization of invasive *Enterobacteriaceae* from pediatric patients in Central and Northwestern Nigeria

**DOI:** 10.1371/journal.pone.0230037

**Published:** 2020-10-26

**Authors:** Carissa Duru, Grace Olanipekun, Vivian Odili, Nicholas Kocmich, Amy Rezac, Theresa O. Ajose, Nubwa Medugu, Dominic Umoru, Chukwuma Onuchukwu, Huda Munir, Binta Wudil Jibir, Zubaida Farouk, Safiya Gambo, Fatimah Hassan-Hanga, Rasaq Olaosebikan, Bernard Ebruke, Charles Esimone, Stephen Obaro

**Affiliations:** 1 International Foundation Against Infectious Diseases in Nigeria, Abuja, Nigeria; 2 Department of Pediatric Infectious Diseases, University of Nebraska Medical Center, Omaha, Nebraska, United States of America; 3 Department of Medical Microbiology and Parasitology, National Hospital Abuja, International Foundation Against Infectious Diseases in Nigeria, Abuja, Nigeria; 4 Maitama District Hospital, Abuja, Nigeria; 5 Federal Medical Centre, Keffi, Nigeria; 6 Aminu Kano Teaching Hospital, Kano, Nigeria; 7 Hasiya Bayero Paediatric Hospital, Kano, Nigeria; 8 Murtala Muhammed Specialist Hospital, Kano, Nigeria; 9 Nnamdi Azikiwe University, Awka, Nigeria; Panstwowy Instytut Weterynaryjny - Panstwowy Instytut Badawczy w Pulawach, POLAND

## Abstract

**Background:**

Bacteremia is a leading cause of mortality in developing countries, however, etiologic evaluation is infrequent and empiric antibiotic use not evidence-based. Here, we evaluated the patterns of ESBL resistance in children enrolled into a surveillance study for community acquired bacteremic syndromes across health facilities in Central and Northwestern Nigeria.

**Method:**

Blood culture was performed for children aged less than 5 years suspected of having sepsis from Sept 2008-Dec 2016. Blood was incubated using the BACTEC^00AE^ system and *Enterobacteriacea* identified to the species level using Analytical Profile Index (API20E^®^). Antibiotic susceptibility profile was determined by the disc diffusion method. Real time PCR was used to characterize genes responsible for ESBL production.

**Result:**

Of 21,000 children screened from Sept 2008-Dec 2016, 2,625(12.5%) were culture-positive. A total of 413 *Enterobacteriaceae* available for analysis were screened for ESBL. ESBL production was detected in 160 *Enterobacteriaceae*, high resistance rates were observed among ESBL-positive isolates for Ceftriaxone (92.3%), Aztreonam (96.8%), Cefpodoxime (96.3%), Cefotaxime (98.8%) and Trimethoprim/sulfamethoxazole (90%), while 87.5%, 90.7%, and 91.9% of the isolates were susceptible to Imipenem, Amikacin and Meropenem respectively. Frequently detected resistance genes were *bla*TEM—83.8% (134/160), and, *bla*CTX-M 83.1% (133/160) followed by *bla*SHVgenes 66.3% (106/160). Co-existence of *bla*CTX-M, *bla*TEM and *bla*SHV was seen in 94/160 (58.8%), *bla*CTX-M and *bla*TEM in 118/160 (73.8%), *bla*TEM and *bla*SHV in 97/160 (60.6%) and *bla*CTX-M and *bla*SHV in 100/160 (62.5%) of isolates tested.

**Conclusion:**

Our results indicate a high prevalence of bacteremia from ESBL *Enterobacteriaceae* in this population of children. These are resistant to commonly used antibiotics and careful choice of antibiotic treatment options is critical. Further studies to evaluate transmission dynamics of resistance genes could help in the reduction of ESBL resistance in these settings.

## Introduction

Bacterial blood stream infection (BSI) is a major public-health concern especially in developing countries as it is one of the leading causes of death. In Nigeria as in most developing countries, sub-standard laboratory methods contribute to improper diagnosis of BSI [1]. The absence of accurate etiologic diagnosis and standard clinical microbiology laboratories results in patients being treated with broad spectrum antibiotics. This unguided approach to clinical care is an important factor which promotes the development and spread of antibiotic resistant bacteria.

Trends of increasing antibiotic resistance of blood pathogens to commonly used antibiotics have been reported in Nigeria [2]. Antibiotic resistance has become a concern worldwide and in *Enterobacteriaceae*, the production of β-lactamase remains the most important mechanism of β- lactam resistance [3].

β-Lactamases are a group of bacterial enzymes that hydrolyze β-lactam antibiotics [4]. The first *β*-lactamases discovered are the broad spectrum TEM-1, TEM-2, and SHV-1. The product of mutations of the genes that encode these enzymes gave rise to current extended-spectrum *β*-lactamases (ESBLs), [4]. The ESBL enzymes were initially recognized in clinical isolates in the 1980s; they are derived mainly from the TEM or SHV types of β-lactamases, by point mutations in the parent enzymes which did not possess extended-spectrum β-lactam substrate activity [5, 6]. More than 200 ESBLs have been identified so far, apart from the TEM, SHV and CTX-M types, other clinically relevant types of ESBLs include the VEB, PER, GES, TLA, IBC, SFO-1, BES-1 and BEL-1 types [6].

Extended-spectrum β-lactamases (ESBLs) are a rapidly evolving group of β-lactamases which hydrolyze the extended-spectrum cephalosporins, penicillins, and aztreonam, but not carbapenems [5]. These ESBL-producing bacteria may also be multiply resistant to other class of antimicrobial agents such as aminoglycosides, trimethoprim/sulfamethoxazole, and quinolones. These Multidrug-resistant (MDR) strains cause infections which are difficult to treat [3, 7, and 8]. The World Health Organization (WHO) has declared infections caused by MDR bacteria as an emerging global health problem of major public health concern [9].

The prevalence of ESBL-producing bacteria has been reported worldwide [10–15], while there are a number of publications on ESBL-producing bacteria causing clinical infections [16–21] in Nigeria, report on the characterization of invasive isolates from infants and children is sparse and because antimicrobial resistance varies greatly among geographical settings, it is crucial to formulate empiric therapy of severe infections such as BSI on comprehensive location specific knowledge of the prevalence and antimicrobial resistance patterns of locally isolated bacteria.

In this study we limited our population to children who were enrolled for community acquired bacteremia surveillance in young Nigerian children. Children are at a greater risk of acquiring bloodstream infections compared to adult due to their immature immune system.[22] We thus aimed to investigate the genetic profile of Extended spectrum betalactamase *Enterobacteriaceae* (ESBL-E) from pediatric BSI patients.

## Methods

### Isolate collection

The study was conducted at seven hospitals in Federal Capital Territory (FCT) and three in Kano Nigeria as previously described from Sept 2008—Dec 2016 [1, 23]. Children aged less than five years were enrolled at the different sites, blood specimens of 1–3 ml were collected using a vacutainer set, after aseptically cleansing the skin with alcohol swab and povidone-iodine, the specimen was collected directly into an aerobic blood culture bottle (BD BactecPeds Plus/F culture vials; Becton Dickinson, Ireland), and incubated in an automated Bactec^®^ 9050 machine. All positive bottles were sub cultured onto MacConkey, Sheep blood and chocolate agar plates at 36°C for 24 h.

A total number of 887 culture-positive *Enterobacteriaceae* were obtained. Of the 887 isolates, 474 salmonella species including *Typhi* which have been reported by Obaro et al. 2015 were excluded [23], therefore 413 including *Escherichia coli*, *Klebsiella* species, *Enterobacter* species, *Serratia marcescens*, *Pantoea* species, *Salmonella Typhi* and *Citrobacter* species from September 2008 to December 2016 were included. The isolates were stored in 10% skim milk glycerol at -80°C.

### Phenotypic screening and confirmation of ESBL

**S**usceptibility was determined using the disk diffusion method on Mueller Hinton agar as recommended by the Clinical and Laboratory Standard Institute [24]. Susceptibility was tested against amoxicillin/clavunate (20/10μg), cefoxitin (30μg), trimethoprim-Sulfamethoxazole (1.25/23.75μg), ciprofloxacin (5μg), ceftriaxone (30μg), amikacin (30μg), cefpodoxime (10μg), ceftazidime (30μg), imipenem (10μg) and meropenem (10μg), cefotaxime (30μg), piperacillin tazobactam (110μg), aztreonam (30μg) and tigecycline (15μg), (Oxoid Ltd, Basingstoke, Hampshire, England) and interpretation of breakpoint was according to CLSI, 2015 guideline.

*Enterobacteriaceae* were identified to species level using Analytical Profile Index (API 20E) identification strip (Biomeriux Inc, France). Phenotypic ESBL production was confirmed with the combination disc diffusion test with clavulanic acid. Confirmatory test was considered positive when the inhibition zone produced by the discs in combination clavulanate increased ≥5 mm than the disks without the clavulanate [24].

### Molecular identification of ESBL genes

All confirmed ESBL bacteria using the combined disk method were analyzed by Real time PCR for the presence of genes encoding TEM, SHV, CTX-M. Primers and Probes were designed for ESBL producing genes by LGC, Biosearch, USA based on primers used by Roschanski *et al*., 2014 [25].

Genomic DNA was extracted using Maxwell 16 cell DNA purification kit (Promega) on an automated DNA extraction machine (Maxwell 16 extraction system, USA). Real time PCR assay was performed on AriaMx system (Agilent Inc, USA) using 25 μL PCR reaction mixture containing 12.5 μL Perfecta master mix low ROX kit (Quanta Bioscience Inc, USA), 1 μL of 10 μM primers, 1 μL of probes, 7.5 μL Nuclease free water (Sigma-Aldrich, USA) and 2 μL DNA template. The thermal conditions were as follows: denaturation at 95°C for 15 min, then 30 cycles consisting of a denaturation step at 95°C for 15 seconds, annealing at 50°C for 15 seconds and extension at 70°C for 20 seconds.

After completion of the run, a cycle threshold (Ct) was calculated by determining the signal strength at which the fluorescence exceeded a threshold limit. This value was analyzed using the AriaMx system software version 3.1.

### Statistical analysis

Two-way ANOVA was performed using SPSS V 21 SPSS Inc., (Chicago, Ill., USA) for statistical analysis of data. A p<0.05 was considered statistically significant.

### Ethical consideration

Consent was obtained from the International Foundation against Infectious Disease in Nigeria (IFAIN), Abuja to gain access to the stored *Enterobacteriaceae* obtained between 2009 and 2016 from blood of children. IFAIN has the written approval by the Research Ethics Committee of the FCT, National Hospital Abuja, Zankli Medical Center, Federal Medical Center Keffi, Aminu Kano Teaching Hospital. Informed consent was obtained from the parent or guardian of the children. All data/sample were fully anonymized.

## Results

From Sept 2008-Dec 2016, 21,000 children were screened for bacteremia with a culture positivity rate of 12.5% (2,625/21,000). Four hundred and thirteen (413) *Enterobacteriaceae* were available for analysis, comprising *Klebsiella* species 141(34.14%), *Escherichia coli* 96(23.24%), *Enterobacter* species 42 (10.16%), *Salmonella typhi* 100 (24.21%) and others 34 (8.23%) (*Serratia*, *Pantoea*, *Citrobacter*, *Proteus* and *Kluyvera* species) (Table 1).

**Table 1:** Frequency of ESBL and Non ESBL producing isolates from blood culture.

Isolates	No of isolates (%)	No of ESBL producers (%)	No of non ESBL producers (%)
*Escherichia coli*	96(23.24)	22 (13.75)	74 (29.24)
*Klebsiellaspp*	141 (34.14)	105 (65.62)	36 (14.22)
*Enterobacter spp*	42 (10.16)	21 (13.12)	23 (9.09)
*Serratia* species	8 (1.93)	4 (2.5)	4 (1.58)
*Pantoeaspp*	14 (3.38)	7 (4.37)	7 (2.76)
*Citrobacters pp*	8 (1.93)	1 (0.62)	7 (2.76)
*Proteus mirabilis*	3(0.72)	0(0)	3(1.18)
*Kluyveraspp*	1(0.24)	0(0)	1(0.39)
*Salmonella typhi*	100 (24.21)	0(0)	100(39.52)
Total (%)	413	160 (38.74)	253 (61.25)

### Prevalence of ESBLs

The overall prevalence of ESBL producers observed in this study was 160/413 (38.74%). Overall, the prevalence of ESBL-producing isolates was greater among male enrollees 53.12% as compared to females 33.12%. Gender was not specified in 13.75%. ESBL-producing *Klebsiella pneumoniae*, *Enterobacter* species, *Pantoea* species and *Serratia* species occurred most frequently in children ≤1 month of age, while *Escherichia coli* was isolated more from age range 2 days -12 months.

**Fig 1:**
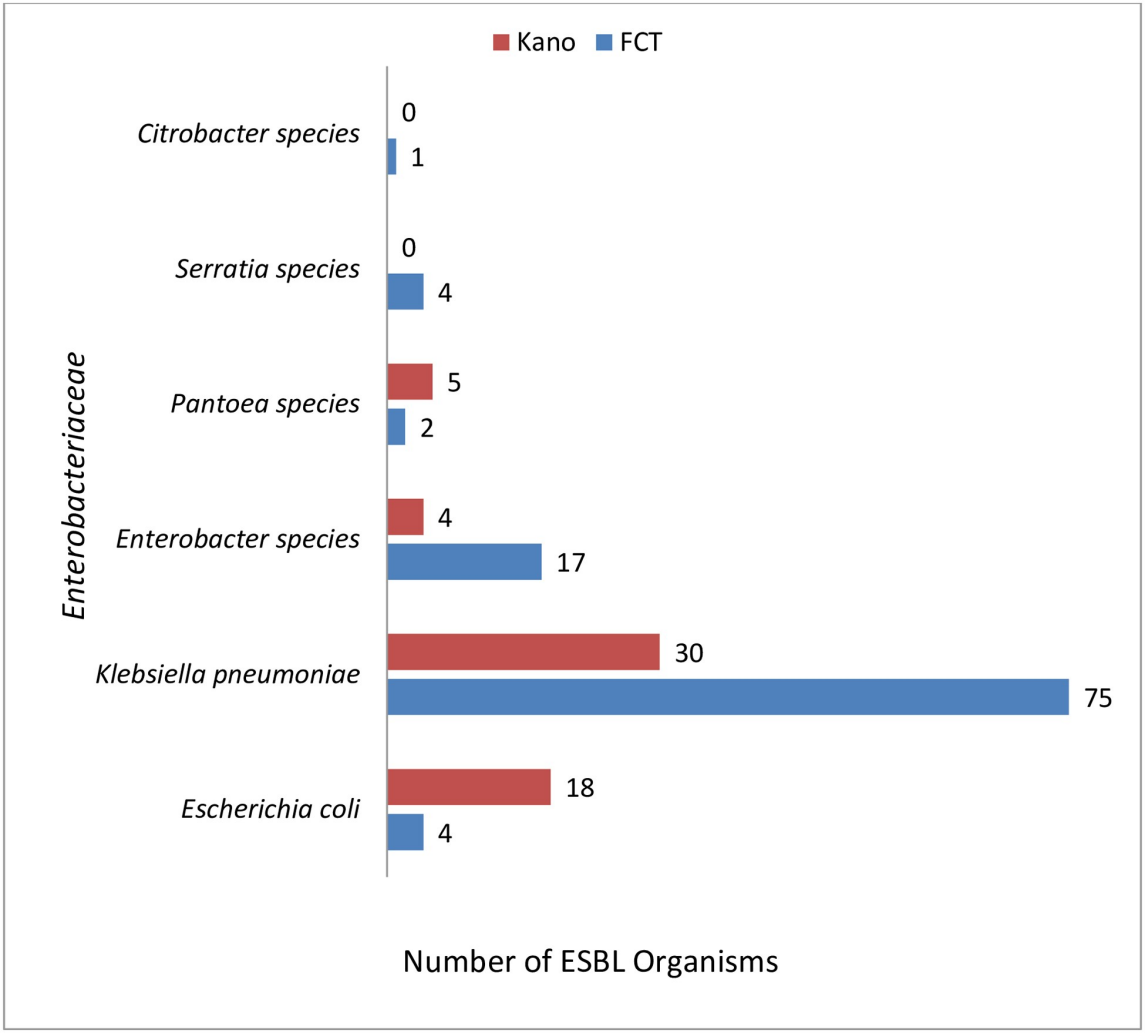
Distribution pattern of ESBL organisms from FCT and Kano.

There was no significant difference (p = 0.813; p>0.05) Fig 1, between ESBL distribution in patients from Kano and those from FCT (FCT 103/160(64.37%) and Kano 57/160 (35.62%)).

### Antibiotic susceptibility data

**Fig 2:**
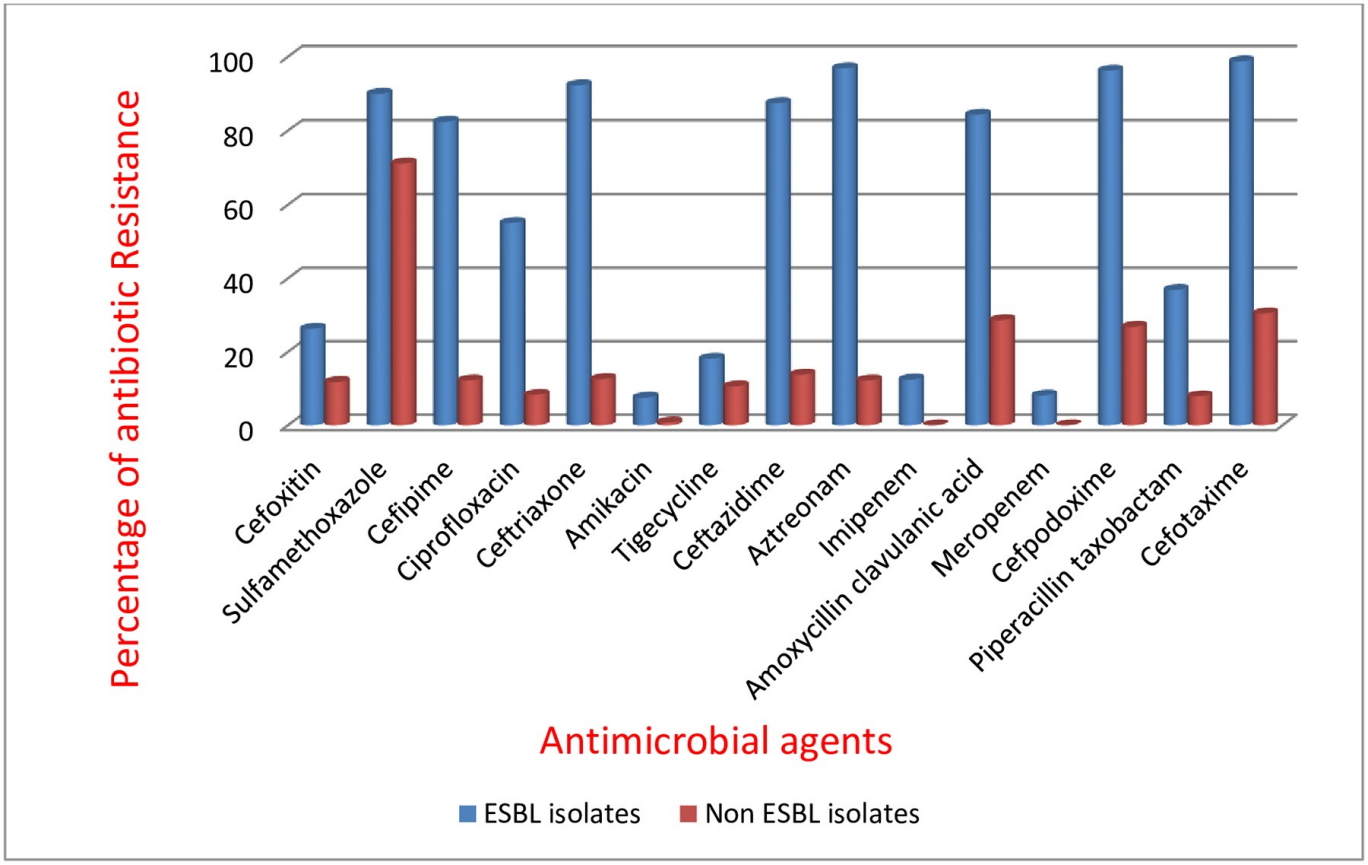
Resistance pattern of ESBL and Non ESBL producing *Enterobactericeae*.

Susceptibility profile of the 160 ESBL-producing isolates revealed high resistance rates for ceftriaxone (92.32%), aztreonam (96.81%), cefpodoxime (96.25%), cefotaxime (98.75%) and sulphamethoxazole- trimethoprim (90%). Over 80% of *the* isolates were resistant to cefepime, Amoxicillin clavulanic acid and ceftazidime (Fig 2), while 87.5%, 90.63%, and 91.87% of the isolates were susceptible to imipenem, amikacin and meropenem respectively.

### *bla* gene composition of ESBL-producing strains

**Fig 3:**
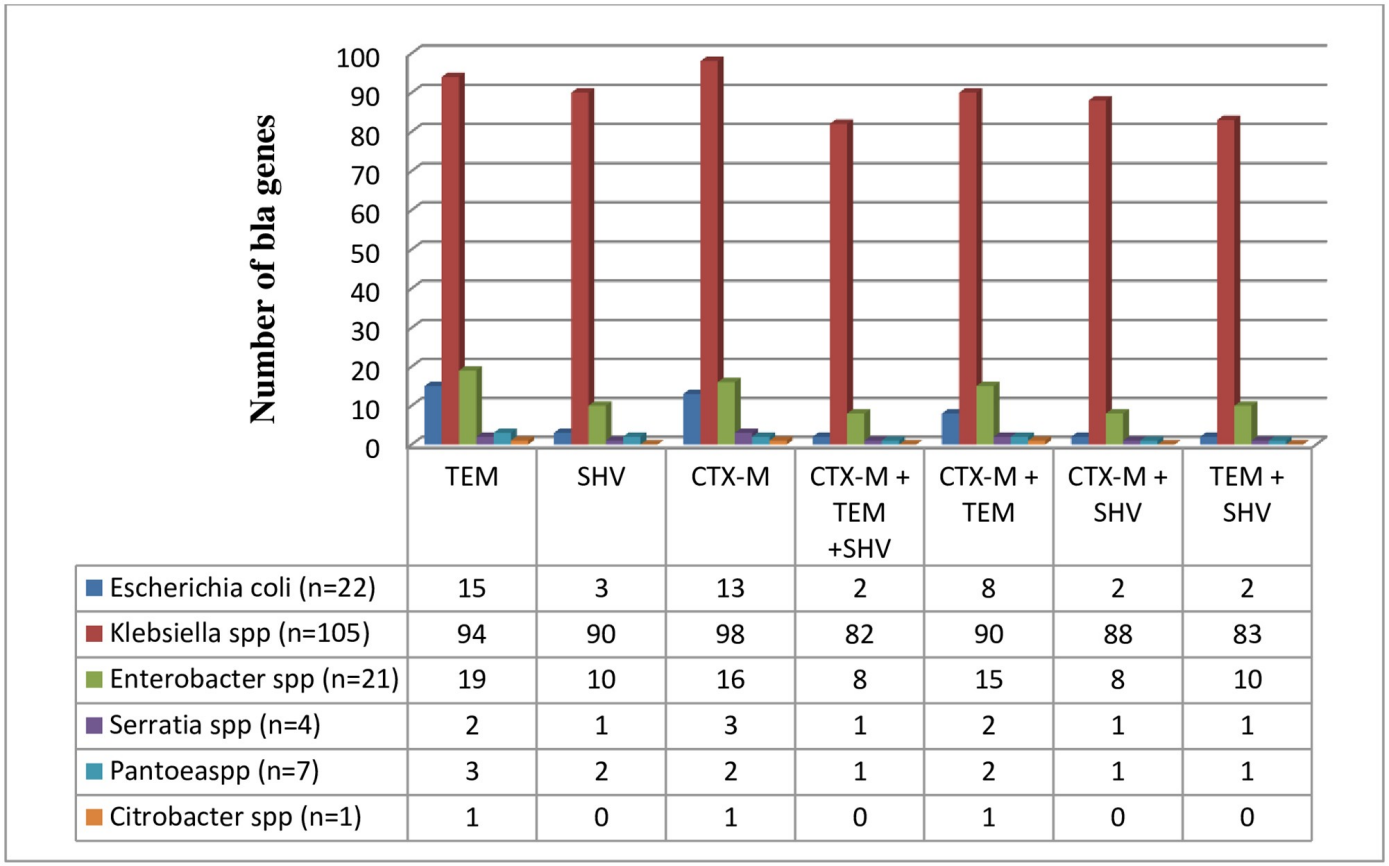
Prevalence of ESBL genes among *Enterobacteriaceae* isolates.

Analysis of the phenotypically confirmed isolates revealed that 134 (62.79%) had the TEM gene, out of which 94(48.14%) were *Klebsiella pneumoniae*, 15 (25.93%) were *Escherichia coli*, 19 (22.22%) were *Enterobacter* species, 2 *Serratia* species, 3 *Pantoea* species and 1 strain of *Citrobacter* species. Also, 133 (34.88%) of the total isolates had the CTX-M gene with 98 (53.33%) being *Klebsiella pneumoniae*, 13 (6.67%) *Escherichia coli*, 5 (33.33%), 16 *Enterobacter* species, 3 were *Serratia* species, 2 *Pantoea* species and one isolate of *Citrobacter* species and 66.25% (106/160) of the total isolates had the SHV gene out of which 90/106 (66.67%) were *Klebsiella pneumoniae*, 3 were *Escherichia coli*, 10 *Enterobacter* species, 2 *Pantoea* species, and1 *Serratia* species (Fig 3). Some of the isolates expressed multiple occurrences of genes, the co-existence of blaCTX-M, blaTEM and blaSHV was seen in 94 of the isolates, while blaCTX-M and blaTEM co-existed in 118 of the isolates, blaTEM and blaSHV in 97 of the isolates while blaCTX-M and blaSHV in 100 of the isolates. Two *Escherichia coli* isolates expressed ESBL phenotype but no blaTEM, blaSHV or blaCTX-M was detected by PCR.

## Discussion

ESBLs have become a widespread serious problem and these enzymes are becoming increasingly expressed by many strains of pathogenic bacteria [26]. The rate of ESBL-producing *Enterobacteriacea* in our study was 38.74% and the isolates were multi drug resistant. ESBL producing isolates showed a higher degree of antimicrobial resistance as compared to non-ESBL producers. Carbapenems and aminoglycosides were shown to be the most effective antimicrobials for both ESBL and Non ESBL isolates. In comparison with non ESBL, there was a significant difference in the antibiotic resistance pattern (P = 0.0004; p>0.05).

From our study, it was observed that there were more ESBL isolates from FCT compared to Kano; this probably might be due to the affordability and access to third generation cephalosporins in FCT. Few studies have investigated the prevalence and genetic characteristics of ESBL producing *Enterobacteriaceae* from blood stream infections of pediatric patients in Nigeria. Kasap et al. 2010 from Southwestern Nigeria reported the isolation of SHV 12 from the blood of a 2 year old girl [27], and Aibinu et al., conducted a study in 2003 in Lagos and found that eight out of 40 *Enterobacter* isolates (20%) investigated were ESBL producers [28]. The limitations in comparison with their studies were the sample size and study period. Their study covered between a period of one month and nine months, while ours had a bigger sample size and a study period of 8 years (September 2008 to December 2016).

In 2012, Alo et al., detected 80% of ESBL production among *Klebsiella pneumoniae* and *Escherichia coli* strains isolated from blood samples of hospitalized patients in Ebonyi State University Teaching Hospital [29]. Also a study by Adeyankinnu et al., 2014 reported an ESBL prevalence of 26.4% for all isolates tested, with *E*. *coli* having a greater proportion [30]. Their study was restricted to detect the presence of ESBL in *Escherichia coli* and *Klebsiella pneumonia*e isolates by phenotypic means only.

The prevalence of ESBL among children in our study was lower when compared to adult studies in Nigeria [20, 31], UK [32], and Spain [33] and pediatric studies in Birmingham [34] and Pakistan [35].

The data from our study have demonstrated that there is a high prevalence of blaTEM, blaCTX-M, and blaSHV ESBL genes in *Enterobacteriaceae* isolates. A study from South Eastern Nigeria reported blaCTX-M-15 genes from urine, vaginal and wound swabs of out-patients younger than 30 years [36]. Two studies conducted in Western Nigeria by Olowe *et al*., [37] from blood, wound, HVS, and sputum samples and Raji *et al*., [38] from blood, Urine and wound samples revealed the isolates harbored blaCTX-M-1 and blaCTX-M-15 genes respectively.

According to our study, blaTEM and blaCTX-M type were the most prevalent ESBL encoding genes, detected in 83% of the ESBL-producing *Enterobacteriaceae* and the majority were found in *Klebsiella* isolates, in contrast to our study, Mohammed *et al*., from North Eastern Nigeria reported blaSHV (36.4%) and blaTEM (31.4%) to be the most prevalent [39], this is likely due to variations in the samples used [39]. The detection of CTX-M, TEM and SHV genes by molecular techniques in ESBL producing bacteria can supply useful data about their epidemiology, association with epidemic clones and risk factors associated with these infections.

The results from our study however, reflect the global trend toward a pandemic spread of CTX-M-type ESBLs in various *Enterobacteriaceae*. These findings agree with other contemporary studies from around the world that show that ESBL genes of the CTX-M are dominant in Tanzania, Burkina Faso, Texas, Spain, Brazil, Latin America, [40–45]. In similarity to our findings, Ahmed et al. [46] reported that blaTEM was the most frequent β-lactamase-encoding gene in Egypt.

In the present study, it was observed that there were multiple occurrences of genes in some of the isolates, this finding is similar to a study in Peru by Garcia *et al*., where majority (57.3%) of the ESBL strains harbored 2 or more ESBL genes [47] while in Turkey, Bali *et al*., observed that about 19.2% ESBL isolates carried more than one type of beta lactamases genes [48].

Two [2] isolates which were screened for ESBL had none of the genes tested for in them from this study; this may be due to the presence of other ESBL genes.

To the best of our knowledge, this is the first report on ESBL resistance patterns from a large surveillance on Bacteremia Pediatric patients from Nigeria.

In conclusion, our findings suggest a high prevalence of ESBL resistance to commonly-used antibiotics in *Enterobacteriaceae* bacteremia in children in this study. Further studies on the transmission dynamics of resistance genes could help in the control of ESBL resistance in these settings.

## Supporting information

S1 File (DOCX)
