# Supplementary material for: Molecular characterization of invasive Enterobacteriaceae from pediatric patients in Central and Northwestern Nigeria
Source: PLoS One. 2020 Oct 26;15(10):e0230037. doi: 10.1371/journal.pone.0230037 (PMC7588054; doi:10.1371/journal.pone.0230037)
Supplement: S1 File — (DOCX) [file pone.0230037.s001.docx]

Supporting Flowchart and Tables.

**Title: Molecular characterization of invasive *Enterobacteriaceae* from pediatric patients in Central and Northwestern Nigeria**

**Authors:**

Carissa Duru PhD, International Foundation Against Infectious Diseases in Nigeria, Abuja, Nigeria. Carissa.duru@ifain.org

Grace M Olanipekun MPH, International Foundation Against Infectious Disease in Nigeria, Abuja, Nigeria [grace.olanipekun@ifain.org](mailto:grace.olanipekun@ifain.org)

Vivian Odili BSc, International Foundation Against Infectious Disease in Nigeria, Abuja, Nigeria odvivian56@gmail.com

Nicholas J Kocmich MPH, Department of Pediatric Infectious Diseases, University of Nebraska Medical Center, Omaha, United States. nick.kocmich@unmc.edu

Amy Rezac MA, Department of Pediatric Infectious Diseases, University of Nebraska Medical Center, Omaha, United States. amy.rezac@unmc.edu

Theresa O Ajose MD, MScPH, International Foundation Against Infectious Disease in Nigeria, Abuja, Nigeria. theresa.ajose@ifain.org

Nubwa Medugu MBBS, FMCPath, International Foundation Against Infectious Diseases in Nigeria, Abuja. nubwa.medugu@ifain.org

Bernard Ebruke MD, International Foundation Against Infectious Disease in Nigeria, Abuja, Nigeria. bernard.ebruke@ifain.org

Charles Esimone PhD, Nnamdi Azikiwe University, Awka, Nigeria. co.esimone@unizik.edu.ng

Stephen Obaro MD, Department of Pediatric Infectious Diseases, University of Nebraska Medical Center, Omaha, United States. stephen.obaro@unmc.edu

**Keywords:** extended-spectrum beta-lactamases, Blood stream Infection, *Enterobacteriaceae*

**Running Title:** ESBL Bacteremia in Neonates in Central and Northwestern Nigeria

**Corresponding Author:**

Carissa Duru PhD

IFAIN, Abuja, Nigeria

Phone : 2348064253456

carissa.duru@ifain.org

**S1 Methodology flowchart**

From Sept 2008-Dec 2016, 21,000 children were screened for bacteremia from CABSYNC and CAPID studies

2,625(12.5%) were culture-positive by automated BACTEC^®^

887 (33.79%) *Enterobacteriaceae* obtained

413 *Enterobacteriaceae* were available for current analysis

474 *Salmonella* species (including S.Typhi) have been reported elsewhere*

Subjected to Antimicrobial susceptibility testing and phenotypically confirmed for ESBL (CLSI, 2015)

Real time PCR for ESBL genes (N=160)

(Roschanski *et al.,* 2014)

* Obaro, S. K et al (2015). Salmonella Bacteremia Among Children in Central and Northwest Nigeria, 2008-2015. *Clinical infectious diseases : an official publication of the Infectious Diseases Society of America*, *61 Suppl 4*(Suppl 4), S325-31.

Community Acquired Bacteremia Syndrome in Young Nigerian Children (CABSYNC)

Community Acquired Pneumonia and Invasive Bacterial Diseases (CAPIBD)

**S2 Primers and Probes for real time PCR amplification of ESBL genes**

Roschanski *et al.,* 2014

| Gene | Primer sequence (5’→ 3’) |
| --- | --- |
| *bla*CTX-M | CTX-M F: CGGGCRATGGCGCARAC |
|  | CTX-M R: TGCRCCGGTSGTATTGCC |
|  | CTX-M P: Yakima Yellow-CCARCGGGCGCAGYTGGTGAC-BHQ1 |
| *bla*TEM | TEM F: GCATCTTACGGATGGCATGA |
|  | TEM R: GTCCTCCGATCGTTGTCAGAA |
|  | TEM P: 6-Fam CAGTGCTGCCATAACCATGAGTGA-BHQ-1 |
| *bla*SHV | SHV F: TCCCATGATGAGCACCTTTAAA |
|  | SHV R: TCCTGCTGGCGATAGTGGAT |
|  | SHV P: Cy5-TGCCGGTGACGAACAGCTGGAG-BBQ-650 |

F= forward primer, R= reverse primer, P = probe

**S3 Prevalence of *bla*TEM gene among ESBL isolates**

| Isolate species | No of ESBL producers | TEM |
| --- | --- | --- |
| *Escherichia coli* | 22 | 15 |
| *Klebsiella* spp | 105 | 94 |
| *Enterobacter* spp | 21 | 19 |
| *Serratia* spp | 4 | 2 |
| *Pantoea* spp | 7 | 3 |
| *Citrobacter* spp | 1 | 1 |
| Total (%) | 160 | 134 (83.75) |

**S4 Prevalence of *bla*SHV gene among ESBL isolates**

| Isolate species | No of ESBL producers | SHV |
| --- | --- | --- |
| *E. coli* | 22 | 3 |
| *Klebsiella* spp | 105 | 90 |
| *Enterobacter* spp | 21 | 10 |
| *Serratia* spp | 4 | 1 |
| *Pantoea* spp | 7 | 2 |
| *Citrobacter* spp | 1 | 0 |
| Total (%) | 160 | 106 (66.25) |

**S5 Prevalence of *bla*CTX-M gene among ESBL isolates**

| Isolate species | No of ESBL producers | CTX-M |
| --- | --- | --- |
| *E. coli* | 22 | 13 |
| *Klebsiella* spp | 105 | 98 |
| *Enterobacter* spp | 21 | 16 |
| *Serratia* spp | 4 | 3 |
| *Pantoea* spp | 7 | 2 |
| *Citrobacter* spp | 1 | 1 |
| Total (%) | 160 | 133 (83.12) |

**S6 Prevalence of co-existence of *bla*CTX-M, *bla*TEM and *bla*SHV genes among ESBL isolates**

| Isolate species | No of ESBL producers | CTX-M + TEM +SHV |
| --- | --- | --- |
| *E. coli* | 22 | 2 |
| *Klebsiella* spp | 105 | 82 |
| *Enterobacter* spp | 21 | 8 |
| *Serratia* spp | 4 | 1 |
| *Pantoea* spp | 7 | 1 |
| *Citrobacter* spp | 1 | 0 |
| Total (%) | 160 | 94 (58.75) |

**S7 Prevalence of co-existence *bla*CTX-M, and *bla*TEM genes among ESBL isolates**

| Isolate species | No of ESBL producers | CTX-M + TEM |
| --- | --- | --- |
| *E. coli* | 22 | 8 |
| *Klebsiella* spp | 105 | 90 |
| *Enterobacter* spp | 21 | 15 |
| *Serratia* spp | 4 | 2 |
| *Pantoea* spp | 7 | 2 |
| *Citrobacter* spp | 1 | 1 |
| Total (%) | 160 | 118 (73.75) |

**S8 Prevalence of co-existence of *bla*CTX-M and *bla*SHV genes among ESBL isolates**

| Isolate species | No of ESBL producers | CTX-M + SHV |
| --- | --- | --- |
| *E. coli* | 22 | 2 |
| *Klebsiella* spp | 105 | 88 |
| *Enterobacter* spp | 21 | 8 |
| *Serratia* spp | 4 | 1 |
| *Pantoea* spp | 7 | 1 |
| *Citrobacter* spp | 1 | 0 |
| Total (%) | 160 | 100 (62.5) |

**S9 Prevalence of co-existence of *bla*TEM and *bla*SHV genes among ESBL isolates**

| Isolate species | No of ESBL producers | TEM + SHV |
| --- | --- | --- |
| *E. coli* | 22 | 2 |
| *Klebsiella* spp | 105 | 83 |
| *Enterobacter* spp | 21 | 10 |
| *Serratia* spp | 4 | 1 |
| *Pantoea* spp | 7 | 1 |
| *Citrobacter* spp | 1 | 0 |
| Total (%) | 160 | 97 (60.62) |
